# A multiparametric advection-diffusion reduced-order model for molecular transport in scaffolds for osteoinduction

**DOI:** 10.1007/s10237-022-01577-2

**Published:** 2022-05-05

**Authors:** Alba Muixí, Sergio Zlotnik, Pere Calvet, Montserrat Espanol, Irene Lodoso-Torrecilla, Maria-Pau Ginebra, Pedro Díez, Alberto García-González

**Affiliations:** 1 International Centre for Numerical Methods in Engineering (CIMNE), Barcelona, Spain; 2 E.T.S. de Ingeniería de Caminos, Universitat Politècnica de Catalunya (UPC), Laboratori de Càlcul Numèric (LaCàN), Barcelona, Spain; 3 Department of Materials Science and Engineering, Group of Biomaterials, Biomechanics and Tissue Engineering, Universitat Politècnica de Catalunya (UPC), Barcelona, Spain; 4 Barcelona Research Centre for Multiscale Science and Engineering, Universitat Politècnica de Catalunya (UPC), Barcelona, Spain

**Keywords:** Reduced-order models, Parametric problems, Scaffold, Proper Orthogonal Decomposition, Bone regeneration, Osteoinduction

## Abstract

Scaffolds are microporous biocompatible structures that serve as material support for cells to proliferate, differentiate and form functional tissue. In particular, in the field of bone regeneration, insertion of scaffolds in a proper physiological environment is known to favour bone formation by releasing calcium ions, among others, triggering differentiation of mesenchymal cells into osteoblasts. Computational simulation of molecular distributions through scaffolds is a potential tool to study the scaffolds’ performance or optimal designs, to analyse their impact on cell differentiation, and also to move towards reduction in animal experimentation. Unfortunately, the required numerical models are often highly complex and computationally too costly to develop parametric studies. In this context, we propose a computational parametric reduced-order model to obtain the distribution of calcium ions in the interstitial fluid flowing through scaffolds, depending on several physical parameters. We use the well-known Proper Orthogonal Decomposition (POD) with two different variations: local POD and POD with quadratic approximations. Computations are performed using two realistic geometries based on a foamed and a 3D-printed scaffolds. The location of regions with high concentration of calcium in the numerical simulations is in fair agreement with regions of bone formation shown in experimental observations reported in the literature. Besides, reduced-order solutions accurately approximate the reference finite element solutions, with a significant decrease in the number of degrees of freedom, thus avoiding computationally expensive simulations, especially when performing a parametric analysis. The proposed reduced-order model is a competitive tool to assist the design of scaffolds in osteoinduction research.

## Introduction

The design of bioinstructive materials has become one of the major challenges in tissue engineering and regenerative medicine. It is based on using different material properties as a language to communicate with cells in order to direct their behaviour, for example, to promote tissue regeneration. Cells are sensitive to different types of stimuli from materials - physical, chemical or mechanical stimuli.

In the field of bone regeneration, one example of the use of bioinstructive materials is material-associated osteoinduction. Some materials have the capacity to direct the differentiation of mesenchymal cells towards the osteoblastic lineage by themselves, without the need to incorporate exogenous growth factors. Several material properties have been identified as playing an important role in this process, such as the microstructure, chemical composition and porosity of the material (Barba et al. 2017, 2018; Barradas et al. 2011).

The mechanisms behind material-associated osteoinduction are not yet fully elucidated. Bohner et al. (2022) propose that the main factors contributing to osteoinduction in bone graft substitutes are the concentration of calcium and/or phosphate ions in vivo, a porosity of the scaffold allowing bone and blood vessel ingrowth, and that the material does not increase the local pH. Among them, ion exchange and, more specifically, calcium (Ca^2+^) and phosphate exchange between the material and the surrounding fluids is one of the factors thought to play a key role (Habraken et al. 2016; Bohner and Miron 2019). The presence of calcium in extracellular fluids controls biomineralisation, i.e. the precipitation of biological apatite on the material surface in vivo, presumably incorporating endogenous proteins, which has been linked to the osteoinductive phenomenon (Habraken et al. 2016). Moreover, Ca^2+^ gradients are directly related to cell migration and growth (Tang et al. 2018). This chemical messenger acts as a “homing signal” that brings together non-differentiated cells for bone remodelling in a specific site and it also triggers differentiation. Indeed, mesenchymal cells are known to respond to calcium and inorganic phosphate levels, which can stimulate their differentiation process into osteoblasts (Chai et al. 2011, 2012; Danoux et al. 2015).

In this context, the architecture of the material, i.e. the size and morphology of the porosity, appears as a very relevant variable, as it is expected to play an important role in the ionic concentration distribution in the surrounding fluids. This may be associated to the finding that osteoinduction is significantly enhanced when the material, that in this context is commonly referred to as a scaffold as it has the role to support bone formation, has concave pores (Habibovic et al. 2005; Ripamonti et al. 2007). Such behaviour was clearly shown in a recent study comparing the capacity of two types of calcium phosphate scaffolds with different pore morphology, namely a foamed and a 3D-printed orthogonal grid of rectilinear strands, to trigger bone formation in a non-osseous site (Barba et al. 2017), whereas significant amount of bone was found in the concave pores of the foamed scaffold, very little bone was formed in the linear pores of the 3D-printed structure. This observation may be the result of very complex phenomena involving cells, growth factors and ions. However, determining ionic transport and distribution of ions through a scaffold geometry is key to clarify the effect that different physical parameters can have on osteoinduction. Numerical simulation offers the possibility to test different scaffold designs and specific properties while reducing experimental efforts, time and cost (Guyot et al. 2014; Van hede 2021; Santamaría et al. 2013).

The transport phenomenon is modelled by an advection-diffusion equation that governs the transport of species in a fluid, for instance, chemical species through a scaffold for “feeding” the cells to differentiate. This leads to computationally demanding parametric models (Spencer et al. 2013), often unaffordable, to be used as a tool for scaffold analysis and design. Reduced-order models are able to drastically lessen such costs, converting these computational models into an affordable tool to support tissue engineering research.

Reduced-order models (ROM) are based on discovering a low-dimensional manifold where the family of parametric solutions lies, and using this information to solve the problem with a significantly lower number of degrees of freedom (Ortega-Gelabert et al. 2020; Díez et al. 2020; Rozza 2014; Pagani et al. 2018). Within ROM techniques, the Proper Orthogonal Decomposition (POD) (Berkooz et al. 1993; Patera and Rozza 2007; Quarteroni and Rozza 2014) is highly appreciated in the scientific community because of its effectiveness and friendly implementation. POD identifies a linear space of reduced dimension by performing a Singular Value Decomposition (SVD) on a representative set of solutions or snapshots. The parametric problem is then solved in the reduced space by means of a Reduced Basis approach.

Here, we propose a parametric model for the distribution of Ca^2+^ in the interstitial fluid across scaffolds. The free parameters account for uncertain input data, with values ranging in relatively large intervals. This allows checking different responses associated with different possible scenarios (flow patterns, diffusion properties...). Two types of realistic scaffolds are considered as benchmarks of application: a foamed and a 3D-printed structured. The scaffold geometry is expected to play a key role in the final concentration of Ca^2+^. Regions in the domain with a high concentration of Ca^2+^ in the numerical solutions are identified as regions where bone is expected to form. The obtained patterns are qualitatively compared to experimental results in the literature (Barba et al. 2017). The number of parameters in the model (six in total) motivates the use of a ROM for multiple evaluation. We study the performance of standard POD and local POD, accounting for both the original snapshots and new, quadratically generated, ones (Díez et al. 2021).

The proposed methodology is a strong tool to get insight on the structural, geometrical and chemical triggers controling bone formation.

The remainder of the paper is organized as follows. In Sect. 2, we state the parametric advection-diffusion problem, present a quantity of interest for analysis and review the reduced-order strategy. Section 3 shows the numerical results, including a comparison between Ca^2+^ patterns in simulations and experimental observations for bone growth, and an exhaustive study for the application of the ROM. The paper is concluded with a discussion and conclusions on the suitability of the proposed methodology for the analysis of scaffolds in tissue engineering in Sect. 4 and 5.

## Materials and methods

**Fig. 1:**
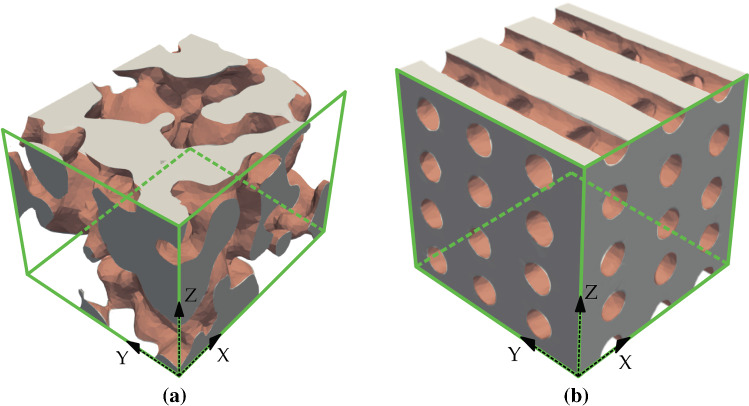
Interstitial domains corresponding to the characteristic volumes for the (a) foamed scaffold and (b) structured scaffold. Green faces indicate the inlet faces, where concentration is set to 0. Light red surfaces correspond to the scaffold-fluid interface where Robin conditions are imposed. The domains are representative volumes of dimensions $750 \times 600 \times 530$ $\mu \text {m}^3$ for the foamed domain, and $1930 \times 2040 \times 1960$ $\mu \text {m}^3$ for the structured one

We aim at defining a reduced-order model for the distribution and concentration of a solute, in our case calcium ions (Ca^2+^), along the interstitial fluid volume in a scaffold geometry. We consider two different realistic geometries, based on a foamed and a structured 3D-printed calcium phosphate scaffolds developed in a previous study, which were tested for osteoinduction by intramuscular implantation in dogs (Barba et al. 2017). The geometries are shown in Fig. 1.

The foamed scaffold has irregular pores with isotropic distribution, with a porosity of $52.3\%$ and a highly variable pore diameter, with an average size of 227 $\mu \text {m}$ as determined by micro-computed tomography. Although using the same material, the structured scaffold corresponds to a 3D-printed model and follows a geometrically structured distribution. In this case, the pores are more regular, with an average pore size of 289 $\mu \text {m}$ and porosity of $54.1\%$ (Barba et al. 2017).

Bone formation is expected to be predominant in those regions with high concentration of Ca^2+^. Using a classical advection-diffusion model, we account for two mechanisms:

- the scaffold releases ions that start filling, by diffusion, the interstitial fluid that goes through the geometry, and
- the fluid drags the ions, thus changing the concentration distribution.

The physical parametrisation of the numerical model is performed by assuming different variations for: *i)* the inflow velocity, regarding its module and direction, *ii)* the fluid viscosity, *iii)* the release rate of Ca^2+^ from the scaffold, and *iv)* a concentration-dependent diffusion coefficient.

Our goal is to assess the influence of the aforementioned parameters to the final distribution of Ca^2+^. The analysis for all possible combinations of parameters, along with the required nonlinear system of equations with a high number of degrees of freedom per each case, motivates the use of a ROM. The proposal presented here is to use a posteriori ROM, following the ideas in Díez et al. (2021).

### Parametric advection-diffusion model

The steady nonlinear advection-diffusion problem for the concentration *c* of Ca^2+^ in a domain $\Omega$, occupied by the interstitial fluid that passes through a scaffold, reads

1 $$\left\{ {\begin{array}{ll} { - {\boldsymbol{\nabla }} \cdot \left( {\nu {\boldsymbol{\nabla }}c} \right) + \boldsymbol{v} \cdot {\boldsymbol{\nabla }}c = 0} \hfill & {{\text{in}}\;\Omega ,} \hfill \\ {c = 0} \hfill & {{\text{on}}\;\Gamma _{I} ,} \hfill \\ {{\boldsymbol{\nabla }}c \cdot \boldsymbol{n} = 0} \hfill & {{\text{on}}\;\Gamma _{O} ,} \hfill \\ { - \nu \frac{{\partial c}}{{\partial \boldsymbol{n}}} = r(c - 1)} \hfill & {{\text{on}}\;\Gamma _{S} ,} \hfill \\ \end{array} } \right.$$

where $\Gamma _I$ is the inlet boundary, $\Gamma _O$ is the outlet and $\Gamma _S$ is the portion in contact with the solid surface, such that $\partial \Omega = \overline{\Gamma _I} \cup \overline{\Gamma _O} \cup \overline{\Gamma _S}$. Here, $\nu := \nu (c,\alpha ,\eta )$ is the diffusivity, ${\varvec{v}}:= {\varvec{v}}(v,\gamma ,\beta )$ is the advection velocity, and ${\varvec{n}}$ stands for the outward unit normal. The coefficient *r* is the release rate of Ca^2+^ from the solid surface.

**Fig. 2:**
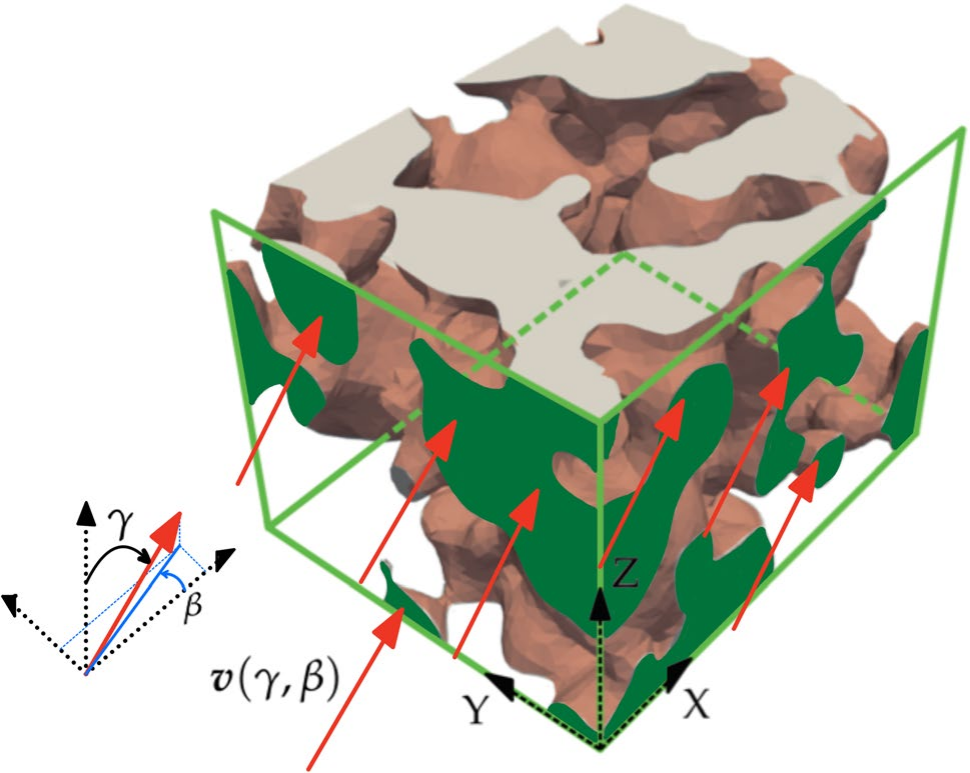
For the domain within the foamed scaffold, the direction of the velocity for the input flow (red arrows) is parametrized with angles $\gamma$ and $\beta$. Green faces indicate the inlet faces, $\Gamma _I$. Opposite faces correspond to the outlet, $\Gamma _O$. The setting is analogous for the structured domain

Dirichlet boundary conditions are set to zero on the inlet $\Gamma _I$ (green areas in Figs. 1 and 2), in order to avoid upstream diffusion against the flow direction. On the outlet $\Gamma _O$, we impose homogeneous Neumann conditions. Finally, on the part of the boundary which is in contact with the scaffold, $\Gamma _S$ (light red surfaces in Figs. 1 and 2), we impose Robin conditions to simulate a release rate of Ca^2+^ from the scaffold towards the interstitial fluid, with rate *r* and up to a saturation concentration of $c = 1$.

Transient effects are not accounted for because we are only interested in the final steady-state distribution of ions.

The parametrisation of the model accounts for six parameters in total. The election of these parameters is discussed next.

- **Inflow velocity field,** ${\varvec{v}}(v, \gamma ,\beta )$. The velocity of the fluid through the scaffold is strongly dependent on several factors, for instance the scaffold location and how it interacts with the surrounding tissue. To the authors’ best knowledge, when a scaffold is implanted, both the flow direction and the velocity module are uncertain, and therefore are taken as parameters to explore their variation. The parametrisation of the angles ($\gamma$ and $\beta$) enables to account for the relative orientation of the scaffold within the surrounding tissues. The resulting molecules distribution might be highly dependent on the flow direction. With this in mind, we propose a parametrisation for the inflow velocity, following the scheme in Fig. 2. The advection velocity ${\varvec{v}}$ is given by a combination of the normalized velocity fields ${\varvec{v}}_x$, ${\varvec{v}}_y$ and ${\varvec{v}}_z$, coming from potential flows in X, Y and Z directions, respectively, that is
  2 $$\begin{aligned} \begin{aligned} {\varvec{v}}(v,\gamma ,\beta )& =v(\sin \gamma \ \cos \beta \ {\varvec{v}}_x + \sin \gamma \ \sin \beta \ {\varvec{v}}_y + \cos \gamma \ {\varvec{v}}_z), \end{aligned} \end{aligned}$$
  with *v* the velocity module, and $\gamma$, $\beta$ the combination angles of the input flow. The module *v* is parametrized in the range [1, 60] $\mu \text {m}/\text {s}$ and $\gamma$, $\beta$ are taken in $[10^\circ , 80^\circ ]$. These ranges have been chosen by numerical experimentation, covering cases in which the final distribution presents highly differentiated patterns.
- **Concentration-dependent diffusivity,** $\nu (c, \alpha , \eta )$. Diffusion modelling is receiving strong attention in this field during last decades. Several models and studies have been developed to elucidate the molecular diffusion process (Spencer et al. 2013; Lee et al. 2013; Karande et al. 2004), from assuming a constant-free diffusion value, to permeability, structure, scale-dependent diffusivities in porous media, or crowding-dependent diffusivities, among others (Tartakovsky and Dentz 2019; Offeddu et al. 2020; Han and Herzfeld 1993). We propose a concentration-dependent diffusivity, for which an increase in the concentration *c* implies a decrease in diffusivity, namely
  3 $$\begin{aligned} \nu (c, \alpha , \eta ) = D_{SE} 10^{-\alpha c}, \end{aligned}$$
  where $D_{SE}$ stands for the constant Stokes-Einstein diffusion coefficient of Ca^2+^ and $\alpha$ is an adimensional parameter taking values in [0, 1]. The value of $D_{SE}$ is obtained through the Stokes-Einstein equation
  4 $$\begin{aligned} D_{SE} = \frac{k_B T}{6\pi \eta \rho }, \end{aligned}$$
  with $k_B =1.38\cdot 10^{-23}$ $\text {JK}^{-1}$ the Boltzmann constant, $T=37^\circ \text {C}$ the temperature, $\rho =114 \text { pm}$ the ionic radius of Ca^2+^ (Manhas et al. 2017) and $\eta$ the viscosity of the fluid (which is also taken as a parameter in the model). The parameter $\alpha$ models the relation between the diffusivity and the existing concentration of ions in the fluid. Note that $\alpha = 0$ implies a constant diffusivity with value $D_{SE}$ in the whole domain, while for $\alpha = 1$ diffusivity is reduced by one order of magnitude in fully crowded regions (where $c = 1$) with respect to regions with no presence of ions (where $c = 0$). For $\alpha > 0$ the problem becomes nonlinear and is solved by applying the Picard scheme with a stopping tolerance of $10^{-8}$ for the relative Euclidean norm.
- **Interstitial fluid viscosity,** $\eta$. The range of values for the fluid viscosity $\eta$ is set to $[5\cdot 10^{-4}, 1.5\cdot 10^{-3}]$ Pa$\cdot$s, which accounts for the range of admissible fluid types. For instance, the viscosity of PBS is $\eta =7\cdot 10^{-4}$ Pa$\cdot$s (Manhas et al. 2017), for deionized water it is $\eta =1\cdot 10^{-3}$ Pa$\cdot$s (Santamaría et al. 2012), and for blood plasma, it takes value in $\eta =1.1-1.3\cdot 10^{-3}$ Pa$\cdot$s (Késmárky et al. 2008). Thus, within the considered range, it is possible to analyse how realistic values of viscosity can modify the distribution of Ca^2+^.
- **Release rate of** Ca^2+^ **from the scaffold,** *r*. The release rate *r* appears in the Robin boundary condition in (1). Note that a higher value of *r* implies a faster release of ions into the fluid. For this analysis, the values of *r* vary in the interval [0.5, 2] m/s, since its extreme values already show high differences in final concentration distributions. The parameter *r* is taken constant, but the model accepts more complex definitions.

Summarizing, the considered parameters are six: the module *v* and the input angles $\gamma$, $\beta$ for the velocity field of the interstitial fluid, the viscosity of the fluid $\eta$, the parameter $\alpha$ defining the relation between the diffusivity and the concentration, and the release rate *r* in the Robin boundary condition on $\Gamma _S$.

Recall that the chosen parameters (and corresponding model behaviour) for this study do not necessary fit with a complex extracellular environment in the scaffold (due to lack of experimental insights). Nevertheless, the main objective here is to show the potential capabilities of the proposed numerical methodology, by using critical parameters in complex scaffolds design studies, with mainly residual computational cost.

### Foamed and structured scaffolds geometry

The described parametric advection-diffusion model is solved for two real scaffolds: foamed and 3D-printed structured, see Fig. 1a and  1b, respectively. The corresponding domains $\Omega$ are the regions through the scaffolds occupied by the interstitial fluid. Green faces indicate the inlet faces on $\Gamma _I$, where the Dirichlet boundary condition $c = 0$ is imposed (as described in Fig. 2), while brown surfaces correspond to the topological scaffold-fluid interface $\Gamma _S$, where the release of ions from the scaffold is modelled by means of a Robin boundary condition.

The 3D finite element models for the two scaffolds were developed from real scaffolds, scanned and segmented by means of a $\mu$-CT and the software 3Dslicer, from which an .stl file of the fluid volumes (inverted from the solid scaffold) were obtained. The final linear tetrahedral (C3D4) meshes were developed using Gmsh for mesh preparation and Abaqus CAE to label the boundaries. For the sake of analysis, a characteristic volume of both foamed and structured scaffold fluid domains were used, with a total of $37\,752$ and $45\,542$ nodes, respectively.

### Quantity of interest: volume ratio

Cell differentiation may occur faster in regions of the domain with a high concentration of ions. As a quantity of intersest (QoI) to compare the performance of the two scaffolds, we measure the percentage of volume of interstitial fluid with a concentration of Ca^2+^ above $90\%$. Since the maximum concentration is $c = 1$ (see the boundary conditions in (1)), this means computing the volume where $c > 0.9$.

To calculate such volume ratio, we take

5 $$\begin{aligned} \text {QoI} = V/V_\Omega \cdot 100, \end{aligned}$$

where $V_\Omega$ is the total volume of the complete domain $\Omega$ of interstitial fluid.

### Reduced Order Models based on Proper Orthogonal Decomposition

In this section, we present an overview of the applied reduced-order methods. The methods are based on a Proper Orthogonal Decomposition (POD) for the family of solutions of the boundary value problem in (1), and follow some of the ideas proposed in Díez et al. (2021). The reader is referred to Díez et al. (2021) for a deeper numerical description of the complete series of methodologies, where also a novel POD strategy based on nonlinear Principal Component Analysis is described (out of scope of the current work).

For the problem at hand, we focus on the well-known standard POD, and its extension to *local* POD (where we account only for local information near the solution) and *quadratic* POD (which incorporates some quadratic terms into the approximation).

#### Standard POD

The finite element discretization of problem (1) leads to a nonlinear system of equations, that is

6 $$\begin{aligned} {\varvec{K}}({\varvec{\mu }}, {\varvec{c}}) \, {\varvec{c}}({\varvec{\mu }}) = {\varvec{f}}({\varvec{\mu }}), \end{aligned}$$

being ${\varvec{c}}\in {\mathbb{R}}^\texttt {d}$ the nodal solution of concentration on the computational mesh, with $\texttt {d}$ degrees of freedom. The input matrix ${\varvec{K}}$, vector ${\varvec{f}}$ and the solution ${\varvec{c}}$ depend on the vector of parameters ${\varvec{\mu }}\in {\mathbb{R}}^{\texttt {n}_{\texttt {P}}}$.

The nonlinear system (6) is solved in a Picard iterative scheme. This consists in subsequently updating an approximation ${\varvec{c}}^{\texttt {old}}$ into an approximation ${\varvec{c}}^{\texttt {new}}$ such that

7 $$\begin{aligned} {\varvec{K}}({\varvec{\mu }},{\varvec{c}}^{\texttt {old}}) \, {\varvec{c}}^{\texttt {new}} = {\varvec{f}}({\varvec{\mu }}), \end{aligned}$$

until convergence.

Recall that here the number of parameters is $\texttt {n}_{\texttt {P}}= 6$, as discussed in Sect. 2.1. Thus, the family of solutions lies in a manifold of dimension six (at most) in the much larger space ${\mathbb{R}}^\texttt {d}$. The idea behind POD is to identify a linear subspace of lower dimension *k* containing the manifold of solutions, with $\texttt {n}_{\texttt {P}}\le k \ll \texttt {d}$, by doing a Principal Component Analysis (PCA) of a representative family of solutions, the so-called *training set*. Afterwards, subsequent solutions are computed in the PCA reduced space, reducing the degrees of freedom from $\texttt {d}$ to *k*.

Consider a sampling of the parametric space ${\varvec{\mu }}^i$ for $i = 1, \ldots , \texttt {n}_{\texttt {S}}$. The corresponding full-order solutions (or *snapshots*) are denoted by ${\varvec{c}}^{i}= {\varvec{c}}({\varvec{\mu }}^{i})$. The snapshots are centered and assembled in a matrix ${\varvec{X}}\in {\mathbb{R}}^{\texttt {d}\times \texttt {n}_{\texttt {S}}}$,

8 $$\begin{aligned} {\varvec{X}}= \left[ {\overline{{\varvec{c}}}}^1\,\, {\overline{{\varvec{c}}}}^2\,\, \ldots \,\, {\overline{{\varvec{c}}}}^{\texttt {n}_{\texttt {S}}} \right] , \end{aligned}$$

with

9 $$\begin{aligned} {\overline{{\varvec{c}}}}^i = {\varvec{c}}^i - \dfrac{1}{\texttt {n}_{\texttt {S}}} \sum _{j=1}^{\texttt {n}_{\texttt {S}}} {\varvec{c}}^{j} =: {\varvec{c}}^i - {\overline{{\varvec{c}}}}. \end{aligned}$$

Centering the snapshots is a standard preprocess to improve the efficiency of PCA.

The PCA, which is based on the Singular Value Decomposition (SVD) of the matrix, is used to eliminate possible redundancies in ${\varvec{X}}$. The SVD leads to

10 $$\begin{aligned} {\varvec{X}}= {\varvec{U}}{\varvec{\Sigma }}{\varvec{V}}^{\textsf {T}} , \end{aligned}$$

where ${\varvec{U}}\in {\mathbb{R}}^{\texttt {d}\times \texttt {d}}$ and ${\varvec{V}}\in {\mathbb{R}}^{\texttt {n}_{\texttt {S}}\times \texttt {n}_{\texttt {S}}}$ are two unit matrices, and ${\varvec{\Sigma }}\in {\mathbb{R}}^{\texttt {d}\times \texttt {n}_{\texttt {S}}}$ is a diagonal matrix of eigenvalues sorted in descendent order, this is, $\sigma _1 \ge \sigma _2 \ge \cdots \ge \sigma _{\texttt {n}_{\texttt {S}}} \ge 0$. The first $\texttt {n}_{\texttt {S}}$ columns of matrix ${\varvec{U}}$, denoted by ${\varvec{u}}^i$, are an orthonormal basis of the linear subspace spanned by the snapshots. The relevant modes in the basis are identified by choosing *k* such that

11 $$\begin{aligned} \sum _{i=1}^k \sigma _i \ge (1-\varepsilon ) \sum _{i=1}^{\texttt {n}_{\texttt {S}}} \sigma _i, \end{aligned}$$

for some tolerance $\varepsilon$. For a new sample in the parametric space, the corresponding solution ${\varvec{c}}$ is then approximated by

12 $$\begin{aligned} {\varvec{c}}\simeq {\overline{{\varvec{c}}}}+ \sum _{i=1}^k {\varvec{u}}^i z^i =: {\overline{{\varvec{c}}}}+ {\varvec{U}}^\star {\varvec{z}}, \end{aligned}$$

with ${\varvec{U}}^\star \in {\mathbb{R}}^{\texttt {d}\times k}$ and ${\varvec{z}}\in {\mathbb{R}}^k$ the new vector of unknowns.

The POD reduced system for ${\varvec{z}}$ is obtained in a reduced basis approach by

13 $$\begin{aligned} \left[ {\varvec{U}}^{\star \textsf {T}} {\varvec{K}}({\varvec{\mu }}) {\varvec{U}}^{\star } \right] {\varvec{z}}= {\varvec{U}}^{\star \textsf {T}} \left[ {\varvec{f}}({\varvec{\mu }}) - {\varvec{K}}({\varvec{\mu }}) {\overline{{\varvec{c}}}}\right] , \end{aligned}$$

which is a system with *k* equations and *k* unknowns.

The standard POD is modified to account for local and quadratic approximations, in order to reduce the computational cost (in the first case) and improve the accuracy of the method (in the latter).

#### Local POD

In local POD, we exploit the idea of the neighbouring snapshots to the new solution including most of the information in (12). “Closeness” is measured in the reduced space with the Euclidean distance in Díez et al. (2021). Here, neighbours are identified in the parametric space ${\mathbb{R}}^{\texttt {n}_{\texttt {P}}}$ in relative Euclidean distance, normalizing the contribution of each parameter by the length of its range.

The POD is performed as usual, but with a local matrix of snapshots ${\varvec{X}}_l$ that only accounts for the $n < \texttt {n}_{\texttt {S}}$ closest snapshots in the parametric space. For a new point ${\varvec{\mu }}$ in ${\mathbb{R}}^{\texttt {n}_{\texttt {P}}}$, we denote by $\mathcal {I}$ the set of indices for these *n* snapshots, this is,

14 $$\begin{aligned} \text {if } i\in \mathcal {I}, \text { then } \Vert {\varvec{\mu }}^i - {\varvec{\mu }}\Vert < \Vert {\varvec{\mu }}^j - {\varvec{\mu }}\Vert \ \forall j\notin \mathcal {I}. \end{aligned}$$

The original matrix of centred snapshots ${\varvec{X}}$ is replaced by

15 $$\begin{aligned} {\varvec{X}}_l = \left[ {\tilde{{\varvec{c}}}}^{i}\, \ldots \right] \ \forall i\in \mathcal {I} , \end{aligned}$$

where the snapshots are centred by subtracting the local average as

16 $$\begin{aligned} {\tilde{{\varvec{c}}}}^{i} = {\varvec{c}}^{i} - \dfrac{1}{n} \sum _{j\in \mathcal {I}} {\varvec{c}}^{j}, \ \forall i\in \mathcal {I}. \end{aligned}$$

Reducing the training set to neighbouring snapshots is intended to improve the computational speed, while retaining most of the accuracy of the POD approximation.

#### Quadratic POD

Quadratic POD is based on the use of an expanded training set, that incorporates quadratic combinations of snapshots. With this, we aim to recover information on the curvature of the manifold of solutions, which may be lost in the linear approximation from standard POD.

The original training set ${\varvec{X}}\in {\mathbb{R}}^{\texttt {d}\times \texttt {n}_{\texttt {S}}}$ is replaced by its expanded version

17 $$\begin{aligned} \begin{aligned}&{\varvec{X}}_q=[{\overline{{\varvec{c}}}}^{1}\,\,{\overline{{\varvec{c}}}}^{2}\,\,\ldots \,\, {\overline{{\varvec{c}}}}^{\texttt {n}_{\texttt {S}}}\,\,\,\,({\overline{{\varvec{c}}}}^{1}\odot {\overline{{\varvec{c}}}}^{1})\,\,\,\,({\overline{{\varvec{c}}}}^{1}\odot {\overline{{\varvec{c}}}}^{2})\,\,\,\,\ldots \,\,\,\, \\&\ldots \,\,\,\,({\overline{{\varvec{c}}}}^{\texttt {n}_{\texttt {S}}}\odot {\overline{{\varvec{c}}}}^{\texttt {n}_{\texttt {S}}-1}) \,\,\,\, ({\overline{{\varvec{c}}}}^{\texttt {n}_{\texttt {S}}}\odot {\overline{{\varvec{c}}}}^{\texttt {n}_{\texttt {S}}})] , \end{aligned} \end{aligned}$$

where $\odot$ stands for the Hadamard product (component by component). The resulting ${\varvec{X}}_q$ has $2\texttt {n}_{\texttt {S}}+ \frac{1}{2} \texttt {n}_{\texttt {S}}(\texttt {n}_{\texttt {S}}-1)$ columns. POD is performed analogously with this new training set.

Local and quadratic approaches can be combined by reducing to neighbouring snapshots first, and then adding their quadratic combinations into the modified training set.

#### Algorithmic description

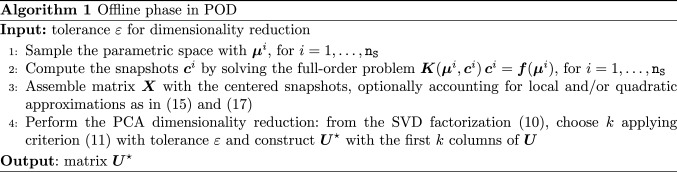

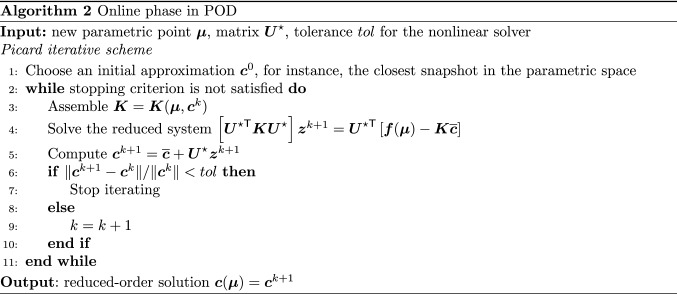





The methodology is summarized for a clearer understanding. The ROM consists of an initial computationally expensive offline phase and a real-time online phase. The offline phase includes the computation of the snapshots and the dimensionality reduction, and is performed once for a sampling of the parametric space and a tolerance $\varepsilon$ in criterion (11), as detailed in Algorithm 1. Then, for each new parametric point ${\varvec{\mu }}$, the reduced-order solution is computed in the online phase, following the steps in Algorithm 2. In this phase, we solve a reduced system at every iteration of the Picard scheme.

## Results

In this section, we present numerical results of the proposed parametric model for the concentration of Ca^2+^ in the foamed and structured scaffold geometries.

First, in Sect. 3.1, we analyse the influence of the chosen parameters in the model in the final distribution of Ca^2+^ ions. Next, Sect. 3.2 shows how the model is able to generate solutions that qualitatively match the experimental results published in the literature for osteoinduction in such type of scaffolds, thus validating our computational approach. In Sect. 3.3, we analyse the accuracy of the ROM as an efficient alternative to evaluate the model. The ROM is finally used to compare the performance of the two scaffolds quantitatively in Sect. 3.4, by computing the volume with a high concentration of Ca^2+^ from reduced-order solutions.

Throughout the section, parameters are expressed in the units in Table 1. All computations are performed using the open-source solver FEniCS (Alnaes et al. 2015; Langtangen and Logg 2017).

**Table 1:** Units and ranges for the parameters in the model

Parameter	Unit	Range
*v*	$\mu$m/s	[1, 60]
$\gamma , \beta$	Degrees	[10, 80]
$\alpha$	–	[0, 1]
$\eta$	Pa$\cdot$s	$[5\cdot 10^{-4}, 1.5\cdot 10^{-3}]$
*r*	m/s	[0.5, 2]

### Influence of parameters

The model in (1) accounts for six parameters, listed in Table 1. Next, we examine the influence of each of the parameters in the pattern of the solution. In order to do so, we take different values for each parameter while keeping the rest of parameters constant.

**Fig. 3:**
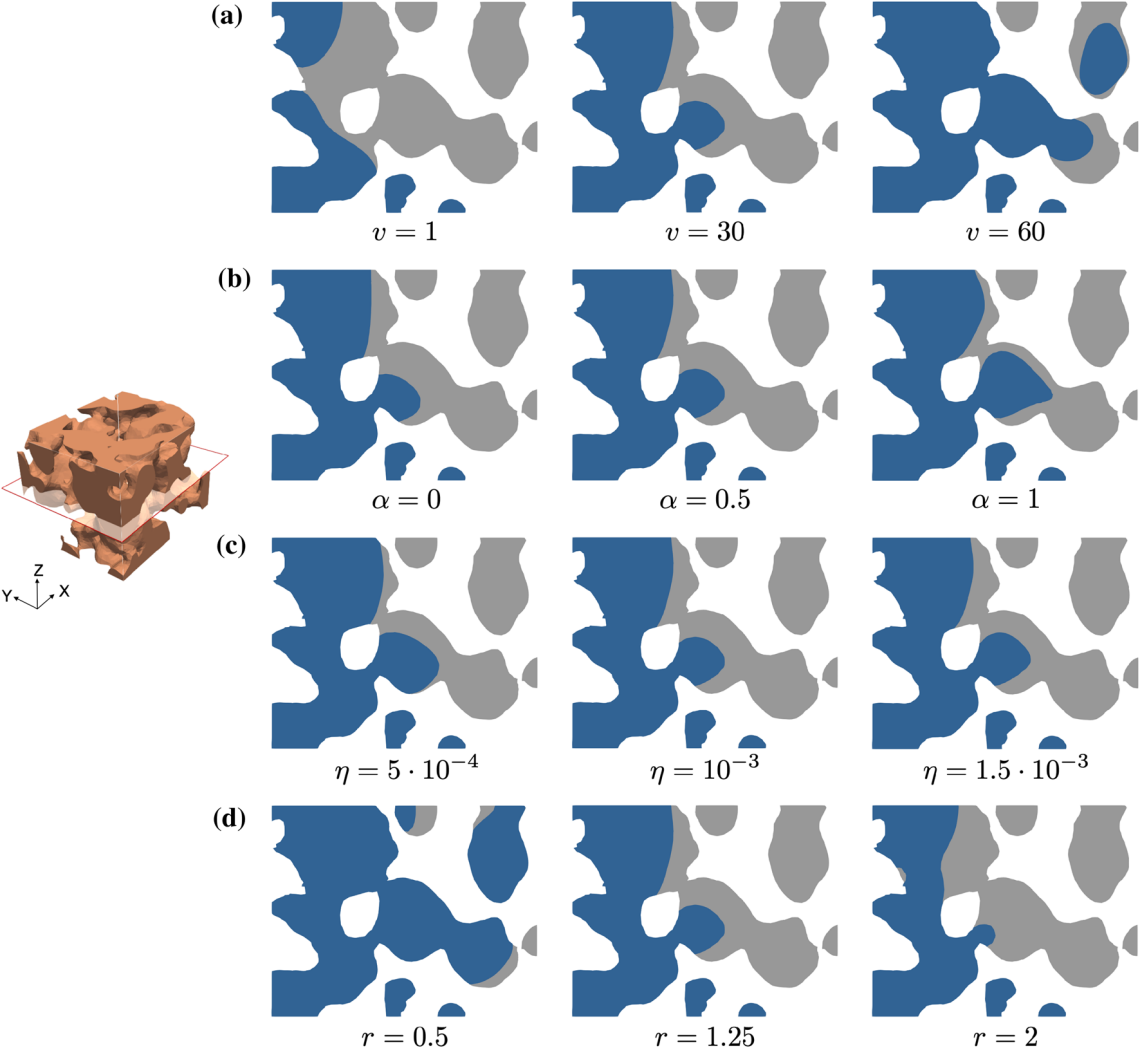
Influence of the parameters in the Ca^2+^ high-concentration region for the foamed scaffold. Solutions correspond to **a** $\alpha = 0.5$, $\eta = 10^{-3}$ , $r = 1.25$, $\beta = \gamma = 45$ for different values of *v*, **b** $v = 30$, $\eta = 10^{-3}$, $r = 1.25$, $\beta = \gamma = 45$ for different values of $\alpha$, **c** $v = 30, \alpha = 0.5, r = 1.25, \beta = \gamma = 45$ for different values of $\eta$, **d** $v = 30$, $\alpha = 0.5$, $\eta = 10^{-3}$, $\beta = \gamma = 45$ for different values of *r*. Regions with a concentration above 0.9 over 1 are plotted in grey, indicating the domain with expected bone formation, in contrast with the dark blue region, where the concentration is below 0.9. Parameters expressed in the units in Table 1

Figure 3 shows the resulting Ca^2+^ high-concentration regions in a cross section for the foamed scaffold. Regions with a concentration above 0.9 over 1 are plotted in grey, indicating the domain with expected bone formation, in contrast with the dark blue region, where the concentration is below 0.9.

**Effect of velocity** *v*. As expected, no bone will appear near the inlet faces because of the entry of clean interstitial fluid that drags the ions across the domain. When increasing the velocity module in the range $v \in [1, 60]$, we observe that the total high-concentrated area diminishes and, therefore, less bone is expected to form, Fig. 3a.

**Effect of** $\alpha$. The parameter $\alpha$, appearing in the diffusivity expression (3), is related to a change in the pattern of bone formation. Recall that for $\alpha = 0$ the diffusivity is constant in the whole domain, while for $\alpha > 0$, diffusivity decreases with concentration. In Fig. 3b, cavities in the distribution become more pronounced for higher $\alpha$, with a thinner layer of bone around the scaffold.

**Effect the viscosity** $\eta$. A slight redistribution of the grey region is observed when increasing the value of $\eta$ in the range $[5\cdot 10^{-4}, 1.5\cdot 10^{-3}]$, see Fig. 3c.

**Effect of the release rate** *r*. With a higher release rate $r \in [0.5, 2]$ the ionic concentration increases faster. This implies a relevant growth of the high-concentration volume in Fig. 3d.

**Fig. 4:**
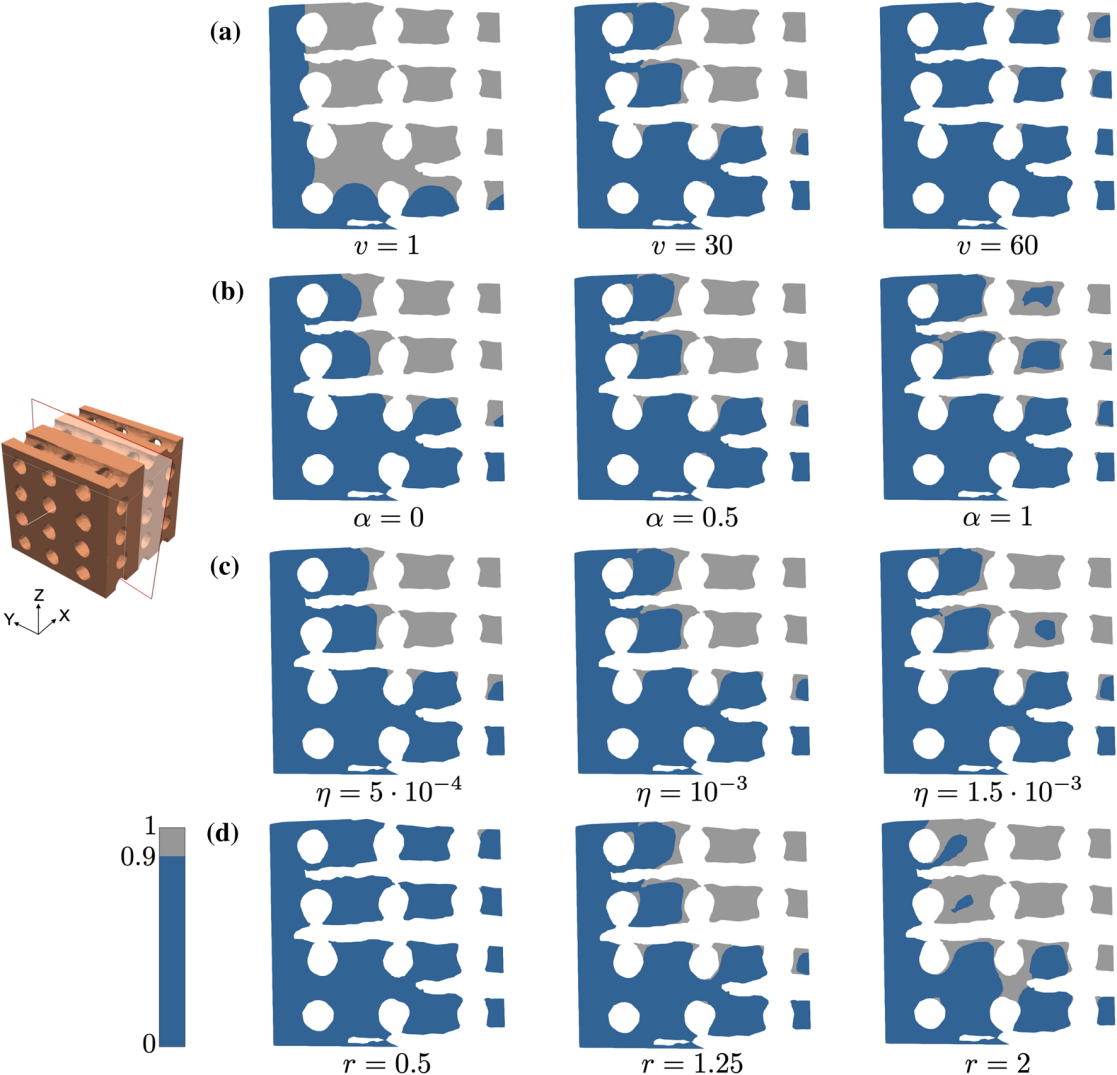
Influence of parameters in the Ca^2+^ high-concentration region for the structured scaffold. Solutions correspond to (a) $\alpha = 0.5$, $\eta = 10^{-3}$ , $r = 1.25$, $\beta = \gamma = 45$ for different values of *v*, (b) $v = 30$, $\eta = 10^{-3}$, $r = 1.25$, $\beta = \gamma = 45$ for different values of $\alpha$, (c) $v = 30, \alpha = 0.5, r = 1.25, \beta = \gamma = 45$ for different values of $\eta$, (d) $v = 30$, $\alpha = 0.5$, $\eta = 10^{-3}$, $\beta = \gamma = 45$ for different values of *r*. Regions with a concentration above 0.9 over 1 are plotted in grey, indicating the domain with expected bone formation, in contrast with the dark blue region, where the concentration is below 0.9. Parameters expressed in the units in Table 1

The same behaviour on varying these parameters can be inferred in the structured-scaffold domain, see Fig. 4. However, in this case, the total volume with a concentration $c > 0.9$ is significantly lower for all simulations.

**Fig. 5:**
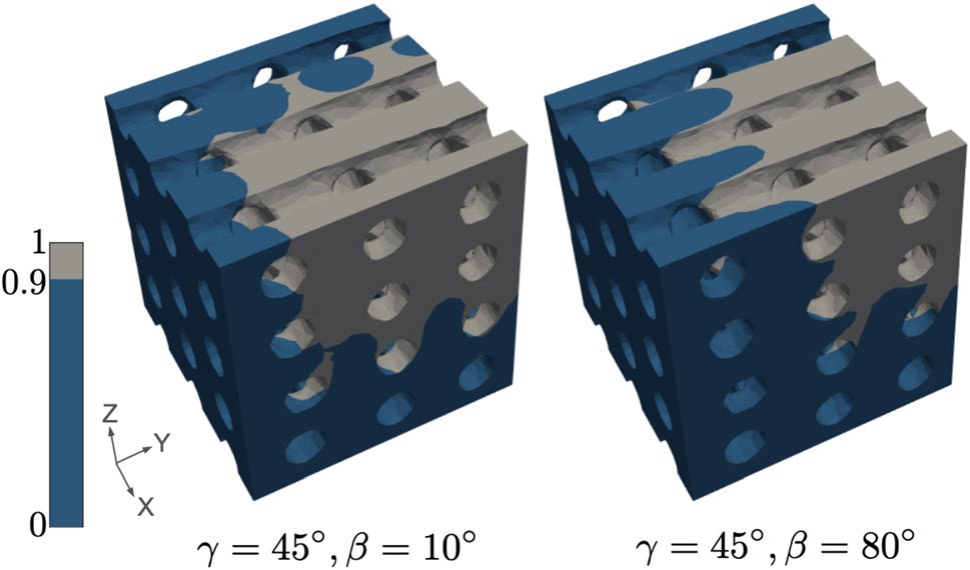
Influence of the inflow direction in the Ca^2+^ high-concentration area for the structured scaffold. Solutions for $v = 30$, $\alpha = 0.5$, $\eta = 10^{-3}$, $r = 1.25$, $\gamma = 45$ for different values of $\beta$. Regions with a concentration above 0.9 over 1 are plotted in grey, indicating the domain with expected bone formation, in contrast with the dark blue region, where the concentration is below 0.9. Parameters expressed in the units in Table 1

As expected, the **input flow angles **$\gamma$ **and **$\beta$ modify the distribution of Ca^2+^ regarding the direction of the deposition. See, for instance, how the variation of $\beta$ changes the solution for the structured scaffold in Fig. 5. However, the characteristics of the deposition (presence of cavities, volume of high-concentration regions) remain similiar.

It is worth mentioning that, in both domains, the chosen parametrisation leads to a rich family of solutions with patterns that cover a wide range of possibilities.

### Model validation: full-order simulations vs experimental observations

We compare numerical simulations from the model in (1) with experimental results by Barba et al. (2017). Assuming that bone formation will be triggered in regions with a high concentration of Ca^2+^ (Tang et al. 2018), we identify the bone patterns around scaffolds that are observed in experiments with those for high-concentration areas in the simulations. For some combinations of the suggested physical parameters, numerical simulations from the proposed model display realistic patterns that are in agreement with those observed in experiments.

**Fig. 6:**
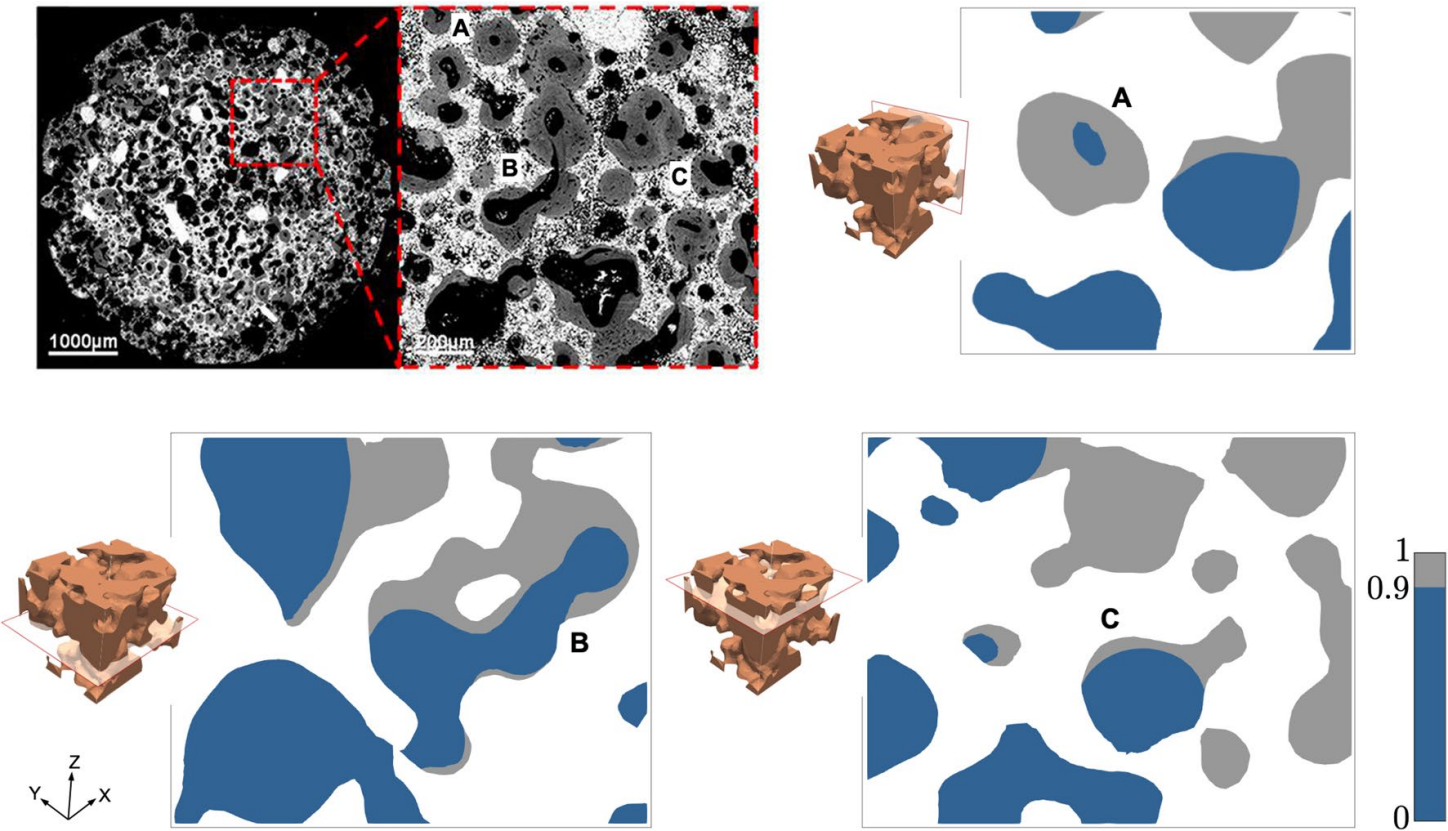
Experimental results obtained by Barba et al. (2017) for a foamed scaffold: black areas correspond to pores (where the interstitial fluid flows), grey to newly-formed bone and white to the scaffold. The numerical simulations correspond to parameters (A) $v = 60$, $\eta = 10^{-3}$, $r = 1.25$, $\alpha = 0.5$, $\beta = \gamma = 45$ and (B and C) $v = 30$, $\eta = 5\cdot 10^{-4}$, $r = 1.25$, $\alpha = 1$, $\beta = \gamma = 45$. In numerical solutions, regions with a concentration above 0.9 over 1 are plotted in grey, indicating the domain with expected bone formation, in contrast with the dark blue region, where the concentration is below 0.9. Parameters expressed in the units in Table 1. Figure adapted with permission from ACS Applied Materials & Interfaces, Volume 9(48), Pages 41722-41736, “Osteoinduction by Foamed and 3D-Printed Calcium Phosphate Scaffolds: Effect of Nanostructure and Pore Architecture” by Barba et al. (2017). Copyright 2017, American Chemical Society

**Fig. 7:**
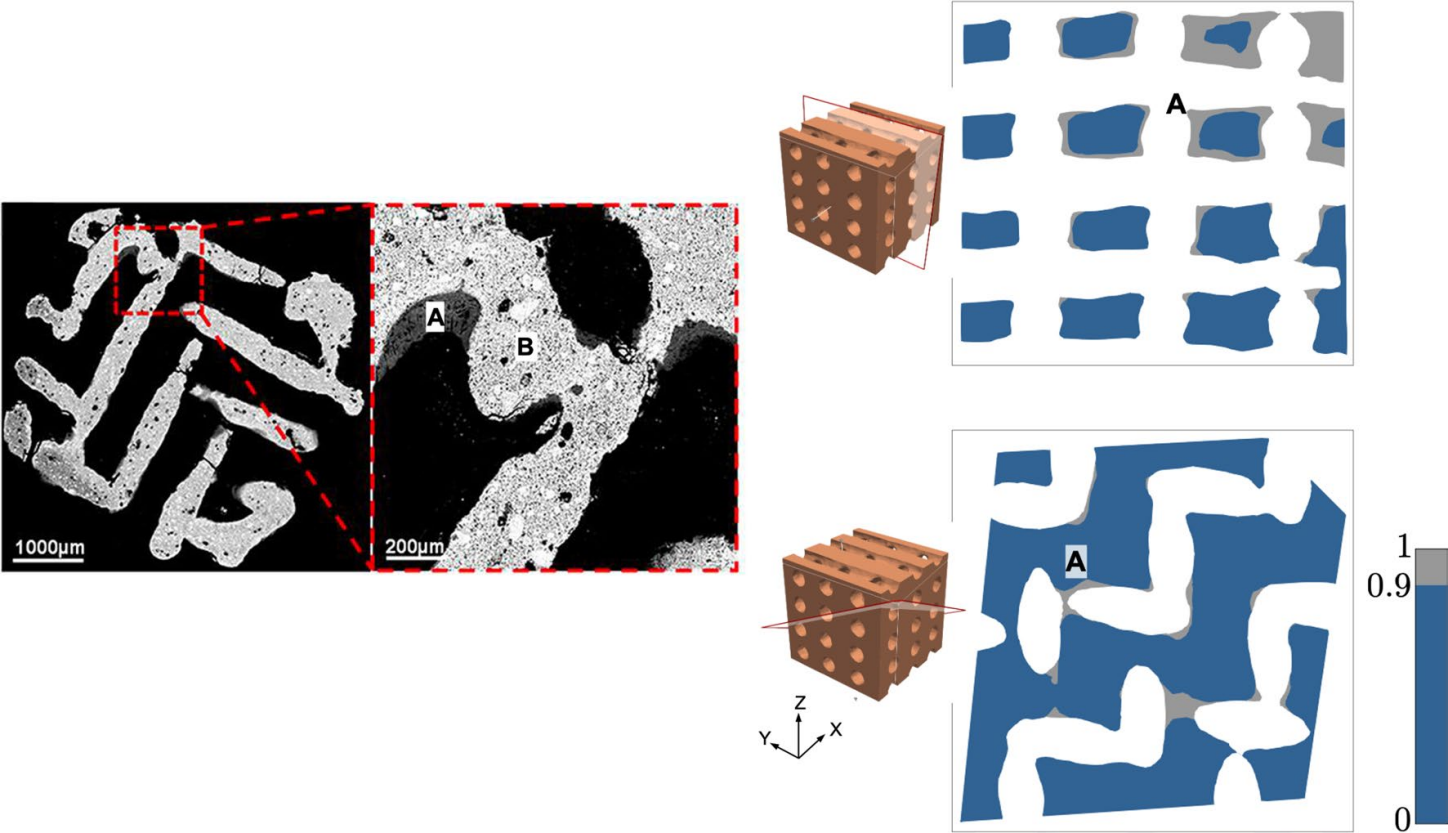
Experimental results obtained by Barba et al. (2017) for a structured scaffold. Bone (**A**) only forms at the intersecting filaments of scaffold (**B**). The numerical simulation corresponds to parameters $v = 30$, $\eta = 10^{-3}$, $\alpha = 1$, $r = 1.25$, $\beta = \gamma = 45$. In numerical solutions, regions with a concentration above 0.9 over 1 are plotted in grey, indicating the domain with expected bone formation, in contrast with the dark blue region, where the concentration is below 0.9. Parameters expressed in the units listed in Table 1. Figure adapted with permission from ACS Applied Materials & Interfaces, Volume 9(48), Pages 41722-41736, “Osteoinduction by Foamed and 3D-Printed Calcium Phosphate Scaffolds: Effect of Nanostructure and Pore Architecture” by Barba et al. (2017). Copyright 2017, American Chemical Society

Our numerical simulations for the foamed scaffold are presented in Fig. 6, together with an experimental image from Barba et al. (2017), where bone formation appears through a foamed scaffold 12 weeks after intramuscular implantation in a canine model. In the image obtained by back-scattered electron microscopy, white areas correspond to the scaffold, grey areas to newly-formed bone and black areas to pores with no bone formation. It is possible to identify three different patterns concerning the growth of new bone. Some pores (marked by A) present a thick and uniform layer of bone around the scaffold. In bottlenecks, the layer becomes thinner or even inexistent (B). Finally, in some pores the layer of bone is irregularly distributed (C). These three patterns can also be seen in our family of simulations, correspondingly marked by A, B and C in Fig. 6.

Analogously, the comparison between experimental and computational results for the structured scaffold is shown in Fig. 7. In this case, Barba and coworkers noticed that bone was only formed at the intersecting filaments of the scaffold. This is also the behaviour we deduct from the Ca^2+^ concentration maps in the numerical results: high-concentration areas are located near intersections, marked by A in the figure.

### POD model evaluation

This section is devised to test the suitability of the presented ROM to approximate solutions for the model in (1). We compare the accuracy obtained with global and local POD, both with linear and quadratic approximations, as described in Sect. 2.4.

In all computations, we set a tolerance $\varepsilon = 10^{-2}$ in criterion (11) to reduce the dimension, which implies keeping a $99\%$ of the accumulated amplitude $\sigma$. Errors for reduced-order solutions are measured using the Euclidean norm with respect to the full-order FE solution.

We consider a coarse training set with 64 snapshots, corresponding to combinations of the extreme values for each parameter. That is, we take all the possible combinations for $v \in \{1, 60\}$, $\beta \in \{10, 80\}$, $\gamma \in \{10, 80\}$, $\alpha \in \{ 0,1\}$, $\eta \in \{ 5\cdot 10^{-4}, 1.5\cdot 10^{-3}\}$ and $r \in \{ 0.5, 2\}$. This training set is used to approximate two new points in the parametric space.

The first point, ${\mathbf {P}}_1$, corresponds to parameters $v = 30$, $\beta = 45$, $\gamma = 45$, $\alpha = 0.5$, $\eta = 10^{-3}$ and $r = 1.25$. The reduced dimensions *k* and the relative errors $E_r$ for the ROM solutions are listed in Tables 2 and 3 for the foamed and structured domains, respectively.

**Table 2:** Foamed scaffold. Reduced dimensions, *k*, and relative errors, $E_r$, for the reduced-order solutions with parameters ${\mathbf {P}}_1$, using a training set with 64 snapshots

	Linear	Quadratic
$n = 64$	$k = 30$ $E_r = 1.24\cdot 10^{-2}$	$k = 222$ $E_r = 8.34 \cdot 10^{-3}$
Local: $n = 20$	$k = 8$ $E_r = 5.64\cdot 10^{-2}$	$k = 19$ $E_r = 3.74\cdot 10^{-2}$
Local: $n = 10$	$k = 5$ $E_r = 6.26\cdot 10^{-2}$	$k = 10$ $E_r = 4.55\cdot 10^{-2}$

**Table 3:** Structured scaffold. Reduced dimensions, *k*, and relative errors, $E_r$, for the reduced-order solutions with parameters ${\mathbf {P}}_1$, using a training set with 64 snapshots

	Linear	Quadratic
$n = 64$	$k = 33$ $E_r = 3.32\cdot 10^{-2}$	$k = 313$ $E_r = 1.83 \cdot 10^{-2}$
Local: $n = 20$	$k = 9$ $E_r = 5.72\cdot 10^{-2}$	$k = 26$ $E_r = 3.65\cdot 10^{-2}$
Local: $n = 10$	$k = 6$ $E_r = 9.72\cdot 10^{-2}$	$k = 13$ $E_r = 6.60 \cdot 10^{-2}$

In both scaffolds, we obtain relative errors of order $10^{-2}$ for the reduced-order approximations, with a significant reduction in the number of degrees of freedom. The most expensive scenario in the considered reduced approximations leads to systems of size $k = 222$ for the foamed scaffold, and size $k = 313$ for the structured one. These only correspond to $0.62\%$ and $0.72\%$ of the degrees of freedom for the full-order problems, respectively.

The differences on the errors between the analysed ROM alternatives are discussed next. First, the relative errors experience a minor decrease when including quadratic approximations, despite the higher computational demands. This behaviour suggests that mechanisms in this problem are simple. Thus, including information on the curvature of the manifold of solutions seems dispensable in this application.

When accounting only for a local approximation, with $n = 20$ or $n = 10$ neighbouring snapshots, we are able to reduce even more the dimension *k* of the problem. As expected, reducing the number of snapshots in the approximation implies an increment in the error of the reduced-order solution. However, errors are still in the same order of magnitude, indicating that the closest snapshots (in the parametric space) include most of the information.

The second point we approximate, ${\mathbf {P}}_2$, takes values $v = 20$, $\beta = 20$, $\gamma = 30$, $\alpha = 0.7$, $\eta = 1.25\cdot 10^{-3}$ and $r = 1.5$. The relative errors are shown in Tables 4 and 5. The obtained values are similar to the ones for ${\mathbf {P}}_1$ and therefore corroborate our observations.

**Table 4:** Foamed scaffold. Reduced dimensions, *k*, and relative errors, $E_r$, for the reduced-order solutions with parameters ${\mathbf {P}}_2$, using a training set with 64 snapshots

	Linear	Quadratic
$n = 64$	$k = 30$ $E_r = 1.38\cdot 10^{-2}$	$k = 222$ $E_r = 9.00 \cdot 10^{-3}$
Local: $n = 20$	$k = 12$ $E_r = 1.60\cdot 10^{-2}$	$k = 56$ $E_r = 1.03\cdot 10^{-2}$
Local: $n = 10$	$k = 7$ $E_r = 2.32\cdot 10^{-2}$	$k = 23$ $E_r = 1.54\cdot 10^{-2}$

**Table 5:** Structured scaffold. Reduced dimensions, *k*, and relative errors, $E_r$, for the reduced-order solutions with parameters ${\mathbf {P}}_2$, using a training set with 64 snapshots

	Linear	Quadratic
$n = 64$	$k = 33$ $E_r = 2.52\cdot 10^{-2}$	$k = 313$ $E_r = 1.53 \cdot 10^{-2}$
Local: $n = 20$	$k = 12$ $E_r = 2.79\cdot 10^{-2}$	$k = 60$ $E_r = 2.19\cdot 10^{-2}$
Local: $n = 10$	$k = 7$ $E_r = 3.75\cdot 10^{-2}$	$k = 24$ $E_r = 2.99\cdot 10^{-2}$

Next, we enrich the training set from 64 to 729 snapshots, accounting for all possible combinations for three equidistant values in each parameter range. In this case, the quadratic approximation of the global POD is discarded for memory requirements. The amount of snapshots generated in ${\varvec{X}}_q$ would be $266\,814$, and the SVD algorithm would require large amount of computer resources to process such a large training set.

Tables 6 and 7 show the errors when approximating ${\mathbf {P}}_2$ for the foamed and structured scaffolds with this new training set. As expected, we observe a gain in accuracy with respect to the coarser training set in all cases, at the price of a higher computational cost.

**Table 6:** Foamed scaffold. Reduced dimensions, *k*, and relative errors, $E_r$, for the reduced-order solutions with parameters ${\mathbf {P}}_2$, using a training set with 729 snapshots

	Linear	Quadratic
$n = 729$	$k = 102$ $E_r = 8.27 \cdot 10^{-3}$	
Local: $n = 20$	$k = 14$ $E_r = 9.14\cdot 10^{-3}$	$k = 52$ $E_r = 8.53\cdot 10^{-3}$
Local: $n = 10$	$k = 8$ $E_r = 1.14\cdot 10^{-2}$	$k = 21$ $E_r = 9.57\cdot 10^{-3}$

**Table 7:** Structured scaffold. Reduced dimensions, *k*, and relative errors, $E_r$, for the reduced-order solutions with parameters ${\mathbf {P}}_2$, using a training set with 729 snapshots

	Linear	Quadratic
$n = 729$	$k = 123$ $E_r = 1.27\cdot 10^{-2}$	
Local: $n = 20$	$k = 14$ $E_r = 1.56\cdot 10^{-2}$	$k = 60$ $E_r = 1.41\cdot 10^{-2}$
Local: $n = 10$	$k = 8$ $E_r = 2.00\cdot 10^{-2}$	$k = 23$ $E_r = 1.74\cdot 10^{-2}$

**Fig. 8:**
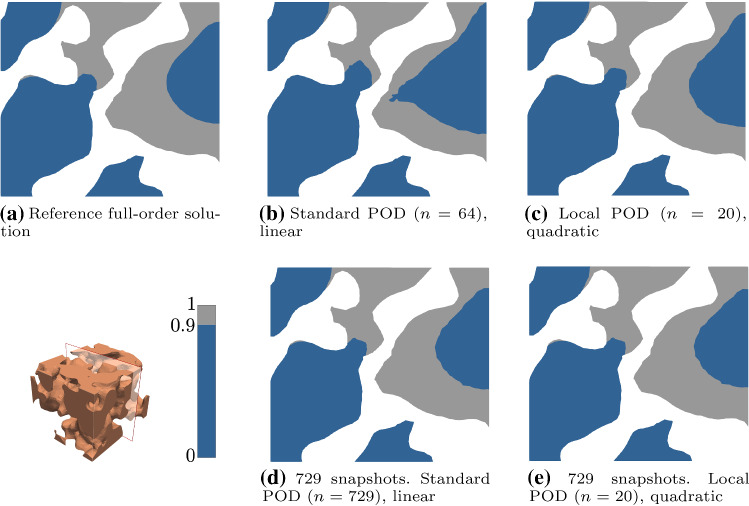
Solutions obtained with different ROM discretizations for parameters ${\mathbf {P}}_2$ ($v = 20$, $\beta = 20$, $\gamma = 30$, $\alpha = 0.7$, $\eta = 1.25\times 10^{-3}$ and $r = 1.5$), using the training sets with 64 and 729 snapshots on the foamed domain. Regions with a concentration above 0.9 over 1 are plotted in grey, indicating the domain with expected bone formation, in contrast with the dark blue region, where the concentration is below 0.9. Parameters expressed in the units in Table 1

**Fig. 9:**
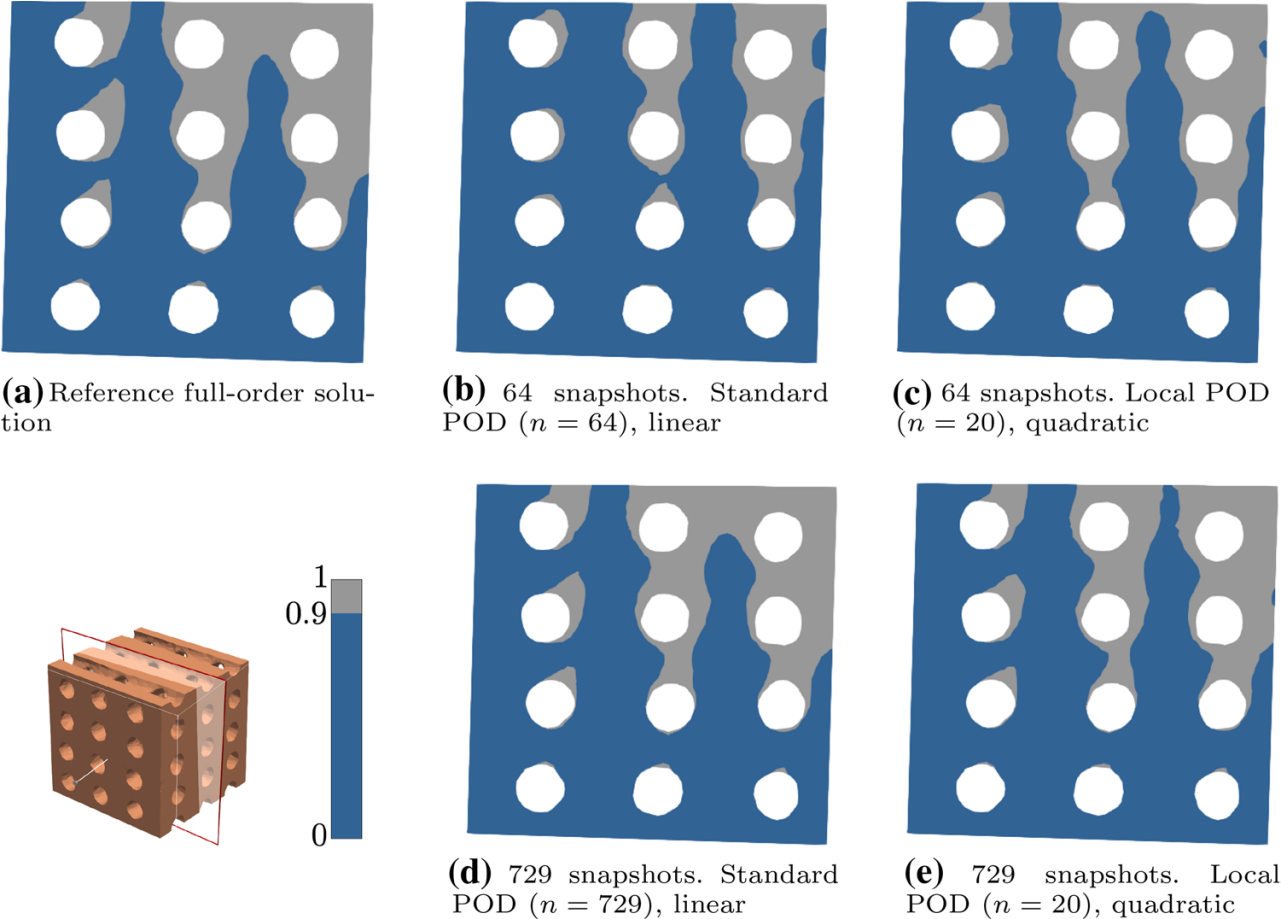
Solutions obtained with different ROM discretizations for parameters ${\mathbf {P}}_2$ ($v = 20$, $\beta = 20$, $\gamma = 30$, $\alpha = 0.7$, $\eta = 1.25\times 10^{-3}$ and $r = 1.5$), using the training sets with 64 and 729 snapshots on the structured domain. Regions with a concentration above 0.9 over 1 are plotted in grey, indicating the domain with expected bone formation, in contrast with the dark blue region, where the concentration is below 0.9. Parameters expressed in the units in Table 1

Finally, for parameters ${\mathbf {P}}_2$, Fig. 8 shows the regions where bone is expected to form in the solutions from the full-order FE, the linear POD and the local-quadratic POD with $n = 20$, in a cross section of the foamed domain for the training sets with 64 and 729 snapshots. We obtain a similar distribution to that from the reference FE solution, with a more evident discrepancy in the pattern for standard POD with the poor training set. Again, from these results we can deduce that enriching the training set improves the accuracy of the reduced-order solution. The incorporation of quadratic terms into the approximation, even for local POD with $n = 20$, seems to improve the quality of the solution, leading to more similar patterns to the reference one. This is also the observed behaviour in the solutions for the structured scaffold domain, see Fig. 9. In this case, the differences on the pattern when taking a global or a local approximation are more notable, but we can also conclude that enriching the training set (with a more dense sampling of the parametric space or by accounting for quadratic contributions) leads to an improvement in accuracy.

The presented results establish the suitability of the ROM techniques in Sect. 2.4 as an efficient option to evaluate the model. The POD performs robustly even though we are dealing with a formulation with six parameters in three-dimensional domains. A representative training set (with enough shapshots) is key to ensure accuracy of the method, especially for local POD.

### Approximation of high-concentration volumes

The goal of this section is twofold. First, we will quantify the variation on the QoI (5) when varying the inflow velocity module *v* and the fluid viscosity $\eta$. Second, we analyse the accuracy on the QoI when using reduced-order solutions for the simulations. In particular, we compare the QoI obtained from the global-quadratic POD solution and the local-quadratic POD solution with $n=10$ with the QoI from the full-order FE solution.

The reduced-order computations are based on the coarse training set with 64 snapshots, corresponding to all combinations for $v \in \{1, 60\}$, $\beta \in \{10, 80\}$, $\gamma \in \{10, 80\}$, $\alpha \in \{ 0,1\}$, $\eta \in \{ 5\times 10^{-4}, 1.5\times 10^{-3}\}$ and $r \in \{ 0.5, 2\}$, expressed in the units in Table 1. The dimension is reduced with a tolerance $\varepsilon = 10^{-2}$ in criterion (11).

**Fig. 10:**
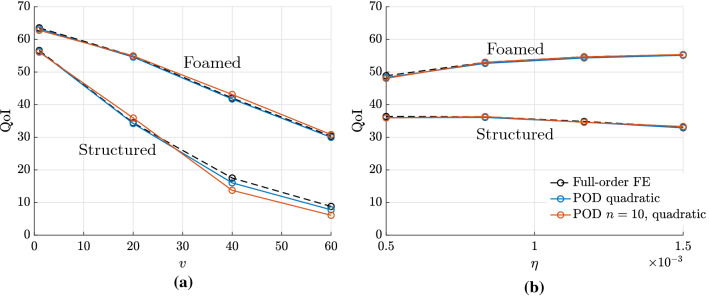
Evolution of the QoI **a** for velocity module $v = 1, 20, 40$ and 60, for $\alpha = 0.7$, $\eta = 1.25\cdot 10^{-3}$, $r = 1.5$, $\beta = 20$, $\gamma = 30$, **b** for viscosiy $\eta = 5\cdot 10^{-4}$, $8.3\cdot 10^{-4}$, $1.17\cdot 10^{-3}$ and $1.5\cdot 10^{-3}$, for $\alpha = 0.7$, $v = 20$, $r = 1.5$, $\beta = 20$, $\gamma = 30$. Parameters expressed in the units in Table 1

Figure 10a shows the evolution of the QoI for solutions corresponding to velocity modules $v = 1$, 20, 40 and 60 $\mu$m/s while keeping the other parameters fixed. For both type of scaffolds, the QoI decreases when increasing the velocity *v*: from a $64\%$ to a $30\%$ in the foamed domain, and from a $57\%$ to an $8\%$ in the structured domain. Note that the QoI is significantly larger for the foamed domain, which is consistent with experimental observations by Barba et al. (2017). The errors on the QoI from the reduced-order solutions are below $1.1\%$ for the foamed case and below $3.8\%$ for the structured one.

We repeat the process, now varying the viscosity $\eta$. The evolution of the QoI for solutions corresponding to viscosities $\eta = 5\cdot 10^{-4}$, $8.3\cdot 10^{-4}$, $1.17\cdot 10^{-3}$ and $1.5\cdot 10^{-3}$ $\text {Pa}\cdot \text {s}$, with the rest of parameters constant, is depicted in Fig. 10b. In this case, the QoI varies slightly: from $49\%$ to a $55\%$ in the foamed domain, and from a $36\%$ to a $33\%$ in the structured domain. As discussed in Sect. 3.1, the value of $\eta$ is related to a different distribution pattern for the concentration rather than a variation in the QoI. In this case, the errors on the QoI are below $0.8\%$ for the POD approximations in the foamed domain, and below $0.4\%$ in the structured one with respect to FE solutions.

The computations for the reduced-order solutions show the right tendency on the evolution of the QoI, which bases the ROM as a potential tool to analyse the parametric performance of scaffolds.

## Discussion

This work offers a tool for numerical simulation in the field of tissue engineering, particularly, in the design of scaffolds for bone regeneration. We propose a parametric advection-diffusion model for the concentration of Ca^2+^ ions in the interstitial fluid across scaffolds, and a numerical data-assisted methodology for the efficient evaluation of the model.

The model accounts for six physical parameters: the module and the angles of the input flow of the interstitial fluid, its viscosity, the release rate of ions from the scaffold and a parameter relating the diffusivity of the ions with concentration. These parameters are considered strongly relevant in reproducing an optimal environment for cell differentiation. By identifying regions with a high concentration of Ca^2+^ with regions where bone is expected to form, we are able to compare numerical results with the outcome in laboratory experiments.

Barba et al. (2017) tested different pore architectures (i.e. pore size and shape) on osteoinductivity using both foamed and 3D-printed calcium phosphate scaffolds. Foamed scaffolds, which are obtained from the solidification of foams, showed higher osteoinductive properties than the structured ones. As a matter of fact, in 3D-printed structured scaffolds, bone formation was mainly reduced to the crossing sections between printed strands, where concavities and tortuosities appear. The results of their work can be seen in Figs. 6 and 7, where we can clearly see that bone formation is predominant for the foamed scaffold. This suggests that a higher structural tortuosity may help to accumulate ions and molecules that induce cell differentiation. The regular geometry of the structured scaffold increases the flow media, and therefore leads to a drastic decrease in bone formation. However, despite the good osteoinduction in foamed scaffolds, one of their main drawbacks is the randomness in the structure creation in foams, with the consequent lack of control over cell differentiation.

The solutions from the proposed model show similar qualitative behaviour to the experimental observations by Barba et al. (2017). Nevertheless, the analysis for the multiparametric problem on a real size scaffold may be computationally unaffordable. Here, we propose to use a reduced-order model technique to drastically reduce the computational cost of the model for the study of scaffolds. In particular, we test the performance of the Proper Orthogonal Decomposition (POD), also accounting for local and quadratic approximations following the work in Díez et al. (2021). The tested POD methods lead to a significant reduction in the number of degrees of freedom for each model evaluation with respect to the full-order FE discretization. In the examples, the number of degrees of freedom is reduced by a factor of, at least, 100. Reduced-order approximations are shown to be robust and accurate.

The model is limited by some simplifications introduced as assumptions. For example, the scaffold is considered as a rigid body, neglecting the effect of its mechanical deformation on the fluid movement. Also, we assume a constant release rate *r*, which instead could be considered as a variable depending on the shear stress of the fluid, the solubility of the material or the cell-associated degradation. Furthermore, it could also be taken into account that body fluids have proteins that could interact with the material and modify ionic release. Nevertheless, the proposed ROM methodology is generalizable to any alternative physical parametrisation required in the design of new scaffolds. Numerical simulations may be used to understand and reinforce experimental results, thus reducing time and costs of in-vivo experimentation in tissue engineering.

## Conclusions

We propose a multiparametric advection-diffusion model for calcium distribution in the interstitial fluid through scaffolds. The model aims to predict bone formation for different scaffolds by the identification of regions with a high concentration of ions. Real-time evaluation of the model, possibly for multiple combinations of physical parameters, motivates the use of Reduced Order Model (ROM) methodologies to drastically reduce the computational cost and quicken the simulations. In particular, we analyse the performance of the Proper Orthogonal Decomposition (POD) with local and quadratic variations. Numerical results are in good agreement with experimental observations reported in the literature, demonstrating the potential of the reduced-order model to efficiently simulate the performance of scaffolds.
